# Monitoring systemic ventriculoarterial coupling after cardiac surgery using continuous transoesophageal echocardiography and deep learning

**DOI:** 10.1007/s10877-025-01328-5

**Published:** 2025-07-17

**Authors:** Jinyang Yu, Tomas Dybos Tannvik, Anders Austlid Taskén, Erik Andreas Rye Berg, Katrine Hordnes Slagsvold, Idar Kirkeby-Garstad, Eirik Skogvoll, Gabriel Kiss, Bjørnar Grenne, Svend Aakhus

**Affiliations:** 1 Department of Circulation and Medical Imaging, Norwegian University of Science and Technology, Trondheim, Norway; 2 Clinic of Cardiology St. Olav’s Hospital, Trondheim University Hospital, Trondheim, Norway; 3 Department of Anesthesia and Intensive Care, St. Olav’s Hospital, Trondheim University Hospital, Trondheim, Norway; 4 Department of Computer Science, Norwegian University of Science and Technology, Trondheim, Norway; 5 Clinic of Cardiothoracic Surgery, St. Olav’s Hospital, Trondheim University Hospital, Trondheim, Norway

**Keywords:** Deep learning, Hemodynamic monitoring, Transoesophageal echocardiography, Mitral annular plane systolic excursion, Left ventricular function, Ventriculoarterial coupling

## Abstract

Deterioration of ventriculoarterial coupling is detrimental to cardiovascular and left ventricular function. To enable continuous monitoring of left ventricular function, we have developed *autoMAPSE*, a new tool that combines transoesophageal echocardiography with deep learning for automatic measurement of mitral annular plane systolic excursion. We hypothesised that autoMAPSE could be used to monitor systemic ventriculoarterial coupling and detect alterations in postoperative cardiac biomarkers. To test this hypothesis, we monitored 50 patients for 120 min immediately after cardiac surgery by measuring autoMAPSE and mean arterial pressure (MAP) every 5 min. Postoperative N-terminal pro B-type natriuretic peptide (ProBNP) and high-sensitivity troponin-T (TnT) were measured twice daily until the evening of postoperative day 1. Ventriculoarterial coupling was assessed non-invasively by calculating arterial elastance and end-systolic elastance (Ea/Ees-ratio). The relationship between autoMAPSE and ventriculoarterial coupling was assessed by 1) correlating Ea/Ees-ratio with one simultaneous autoMAPSE measurement, and 2) relating the measurements of autoMAPSE with corresponding MAP within each patient using a linear mixed model with random slopes. We found that autoMAPSE correlated negatively with Ea/Ees-ratio (*rho* = − 0.61, *P* < 0.05). Furthermore, the individual slopes relating autoMAPSE to MAP were highly significant (*P* < 0.001) and markedly heterogeneous (both positive and negative), suggesting that ventriculoarterial coupling differs substantially in different individual patients. Finally, continuous autoMAPSE measurements were negatively correlated with both peak postoperative ProBNP (*rho* = − 0.46, *P* < 0.001) and TnT (*rho* = − 0.29, *P* < 0.05). In conclusion, continuous monitoring using autoMAPSE in the first two postoperative hours reflected ventriculoarterial coupling as well as peak ProBNP and TnT during the subsequent 24 h.

**Graphical abstract:**

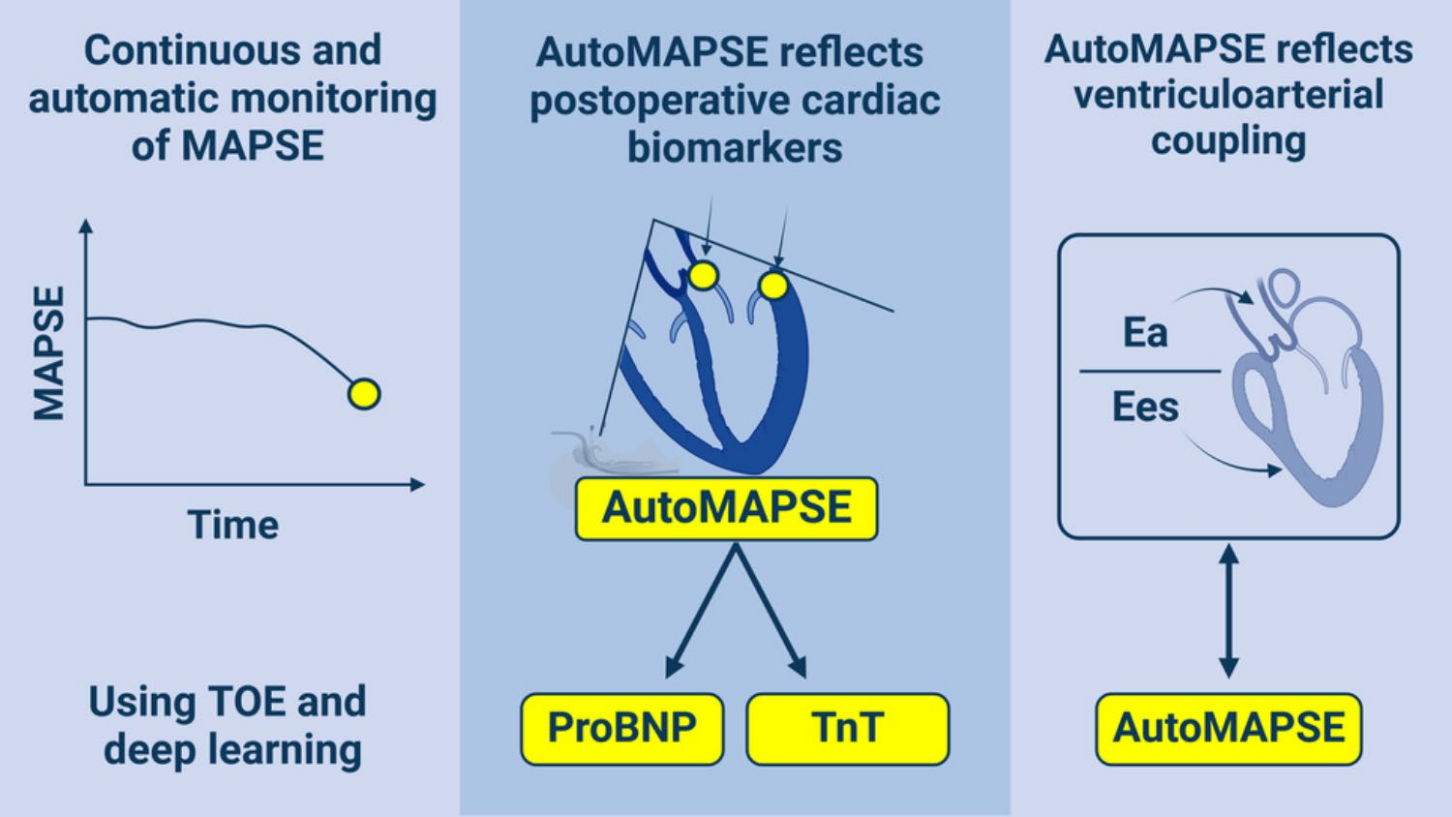

**Supplementary Information:**

The online version contains supplementary material available at 10.1007/s10877-025-01328-5.

## Introduction

Optimizing hemodynamic support is sometimes challenging when caring for patients after cardiac surgery. Postoperative hemodynamic changes, common and often unavoidable, can affect left ventricular (LV) load and function at unpredictable times [1, 2]. When these interactions become pathological, they can manifest as systemic ventriculoarterial (VA) uncoupling and LV dysfunction [3, 4]. Untreated, the heart releases biomarkers like N-terminal pro B-type natriuretic peptide (ProBNP) and high-sensitivity troponin-T (TnT). As expected, patients with postoperative increases in these biomarkers have a worse outcome [5–9].

Continuous monitoring of LV function offers opportunities for earlier therapeutic adjustments, potentially before biomarkers are released. Unfortunately, current assessments of LV function are far too slow and intermittent for effective continuous monitoring [10]. Therefore, detection of LV dysfunction often occurs after complications have entered. Thus, we have developed [11], validated [11, 12], and refined [13] a new method for continuous monitoring of LV function in perioperative patients. This method, i.e., *autoMAPSE*, combines 2D transoesophageal echocardiography (TOE) with deep learning to automatically measure mitral annular plane systolic excursion (MAPSE). As autoMAPSE recently demonstrated excellent feasibility for continuous monitoring [14], the next step is to explore its clinical usefulness.

Since MAPSE depends on both LV load and contractility, we hypothesized that changes in autoMAPSE measurements reflect alterations in systemic VA coupling, and moreover, that continuous autoMAPSE measurements could detect the subsequent release of cardiac biomarkers. Thus, this study aimed to determine, firstly, the relation between autoMAPSE and VA coupling, and secondly, the association between continuous autoMAPSE measurements and the subsequent peak postoperative ProBNP and TnT.

## Methods

### Patients

In this prospective observational study, 50 patients scheduled for on-pump cardiac surgery with a clinical indication for intraoperative TOE were included between October 2022 and March 2023 at St. Olav’s Hospital, Trondheim University Hospital, Norway. The exclusion criteria were age less than 18 years old, unwillingness to participate, and clinical contraindications to TOE [15]. Data on the same patient material has been previously published in studies with unrelated objectives to this present study [13, 14, 16, 17].

This present study conforms with the principles of the Declaration of Helsinki and was approved by the Regional Committee for Ethics in Medicine (REK number 2022/345556). All patients provided written informed consent before participating.

### Continuous monitoring of mitral annular plane systolic excursion

We used autoMAPSE to monitor each patient for approximately 120 min postoperatively while they were still mechanically ventilated, starting immediately after they arrived at the intensive care unit. The methodology, feasibility, and precision of continuous monitoring using autoMAPSE in 2D images were recently described in detail [14]. Briefly, the TOE probe was stabilized by a probe-holder, which allowed hands-free image acquisition for continuous monitoring. The probe tip was always unlocked to minimize the risk of oesophageal trauma. Every five minutes, a set of hands-free images of the midoesophageal two-chamber (2C) view was recorded; each set comprised ten heartbeats. These images were focused on the mitral annulus. For consistency, the imaging plane was predefined at 90 degrees, and the image sector was maximized. The probe position was optimized every 20 min to achieve consistent image quality. Outside each scheduled 20-min interval, the probe was adjusted only if the left ventricle was completely displaced from the image. A 6VT-D probe (GE Healthcare, Horten, Norway) and an E95 ultrasound scanner (GE Healthcare, Horten, Norway) were used for all recordings.

The pipeline of autoMAPSE comprises a deep learning convolutional neuronal network trained under supervision to detect the mitral annulus in 2D TOE images [13] and a set of filtering algorithms to reject erroneous measurements [11]. The deep learning network behind autoMAPSE has been described in detail previously [13]. To summarize, this network first detects the mitral annulus in each echocardiography frame. Next, it measures MAPSE as the longitudinal distance travelled by the annulus. Before reporting MAPSE, a set of filtering algorithms assesses each heartbeat for feasibility, where the criteria for rejection of a specific heartbeat were: 1) if there was a frame-to-frame movement of the mitral annulus > 5 mm, 2) if the mitral annulus was undetectable in less than 60% of the frames per heartbeat, and 3) if the highest position of the mitral annulus did not correspond to the R-wave of the electrocardiogram [11]. The final output is the MAPSE of the two LV walls per recording, reported in millimetres. In this study, we only report the measurement from the anterior wall because this wall has repeatedly demonstrated the highest feasibility [12, 14]. AutoMAPSE works in real-time and can average many measurements within a few seconds, which minimises the effects of ventilatory-induced variability in LV load by comprising the entire ventilatory cycle, which in turn improves the measurement precision, and ultimately allows the detection of smaller changes in MAPSE [12, 14]. Thus, unless stated otherwise, all our measurements using autoMAPSE are the average of up to ten consecutive heartbeats.

### Commonly monitored hemodynamic variables

Every five minutes, we also recorded invasive radial artery blood pressures (systolic, diastolic, and mean arterial pressure [MAP]), heart rate, and central venous pressure (CVP); the corresponding rate-pressure product (RPP) was calculated as the systolic arterial pressure times the heart rate. To assess common surrogates of tissue perfusion, we obtained a set of arterial and central venous blood gases every 40 min and measured lactate, central venous oxygen saturation (ScvO_2_), and central veno-arterial CO_2_-difference (PcvaCO_2_).

### Calculating ventriculoarterial coupling

**Fig. 1:**
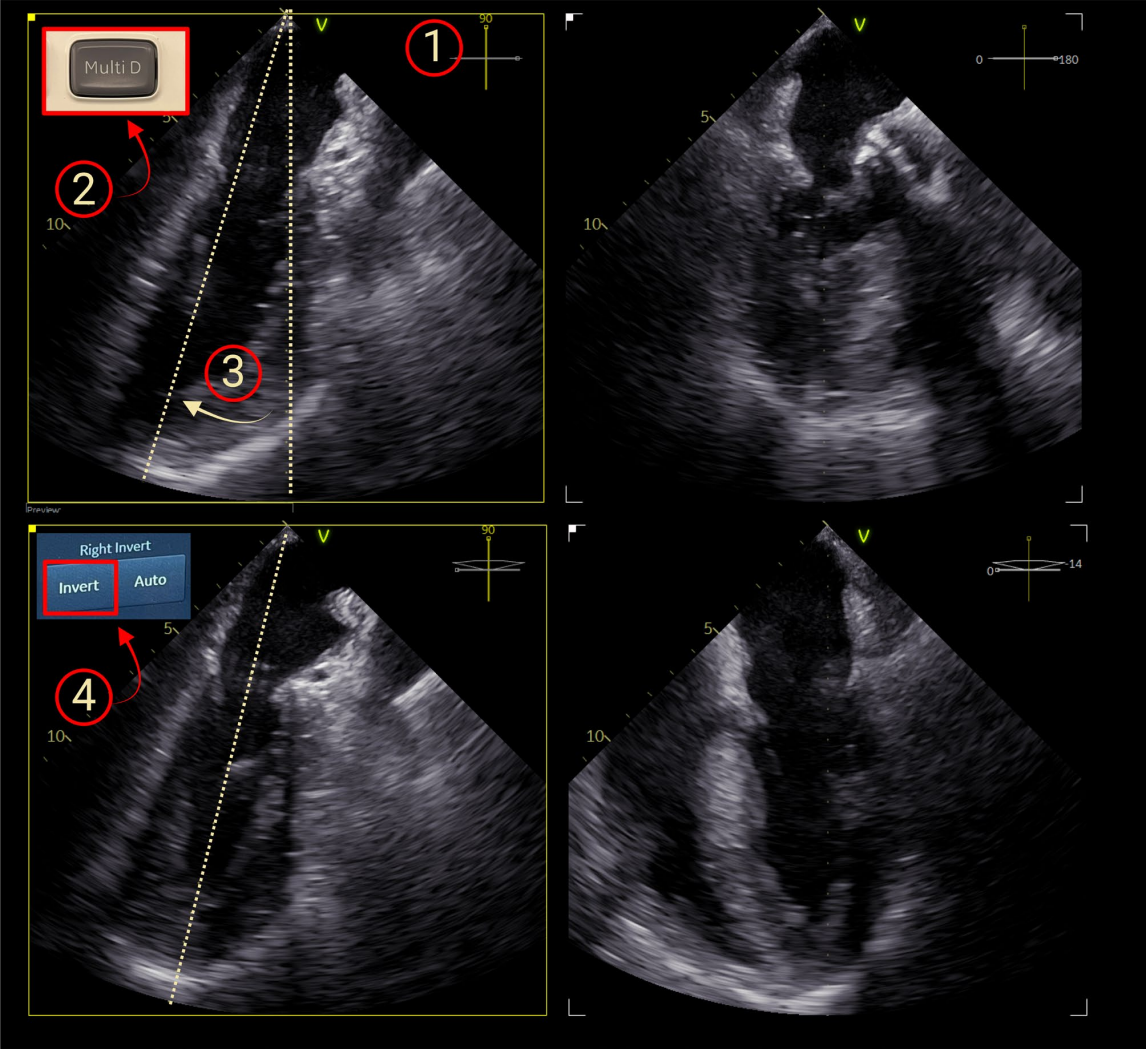
Foreshortening of midesophageal images was minimized by the following steps. **1.** Retroflecting and turning the probe with the omniplane at 90 degrees to acquire a midesophageal two-chamber view with the longest left ventricular length. **2.** Activating simultaneous biplane imaging (Multi-D®, GE Healthcare, Horten, Norway). **3.** Aligning the secondary sector through the left ventricular apex. **4.** Inverting the secondary sector and slight rotation of the omniplane to avoid the left ventricular outflow tract, thus acquiring an optimized midesophageal four-chamber view

VA coupling was assessed as the ratio between effective arterial elastance (Ea) and end-systolic elastance (Ees) [4], where Ees was calculated using the non-invasive method described by Chen et al [18]. For this method, stroke volume (SV) was measured by pulsed-wave Doppler from the LV outflow tract, obtained in either the transgastric long-axis view or a deep transgastric view, the systolic ejection time and pre-ejection time on the same Doppler signal, and the systolic and diastolic arterial pressures simultaneously obtained from a radial artery catheter. The LV outflow tract diameter was measured in a midoesophageal long-axis view. In the case of aortic valve prosthesis, the LV outflow tract diameter was measured just proximal to the sewing ring of the prosthesis [19]. Left ventricular ejection fraction (LVEF) was measured using Simpson’s biplane method of discs [20]. To minimize the foreshortening of the midoesophageal four-chamber view (4C), the midoesophageal images were optimized using simultaneous biplane imaging (Multi-D®, GE Healthcare, Horten, Norway) where the temporal resolution was increased to at least 40 frames per second (Fig. 1). Ea was calculated as 0.9 times the systolic arterial pressure divided by SV [4]. Due to the need for stable hemodynamics, the measurements were obtained at the end of the monitoring period, except in 4 (8%) patients, of whom the measurements were obtained at the beginning. Hemodynamic stability was defined as a stable MAP, heart rate, and drug infusions during data recording. AutoMAPSE and manual MAPSE [11] were also measured in the same B-mode images. All these measurements were the average of three to five consecutive heartbeats and were performed in EchoPAC software (version 204, GE Healthcare, Horten, Norway).

### Cardiac biomarkers

We measured ProBNP and TnT preoperatively and at four time-points postoperatively: 1) immediately after ICU arrival, 2) in the evening on the day of surgery, 3) morning of postoperative day 1, and 4) evening of postoperative day 1. Both biomarkers were analysed immunologically using Cobas 8000 (Roche Diagnostics, Norway), and the established within-lab coefficient of variation was 2.9% for ProBNP and 4.0% for TnT.

### Statistical analysis

We assessed the relationship between autoMAPSE and VA coupling using two approaches. First, we calculated Spearman’s non-parametric correlation coefficient *rho* between Ea/Ees-ratio and the corresponding autoMAPSE measurement. In the second more indirect approach, we explored how MAP affected autoMAPSE by fitting a linear mixed effects model that related autoMAPSE (dependent variable) to MAP (predictor variable), including *patient* as the random factor with both a random intercept and a random slope for the relation. In this model, patient heterogeneity was quantified by the intraclass correlation.

The repeated measurements of autoMAPSE, MAP, CVP, heart rate, RPP, ScvO_2_, PcvaCO_2,_ and lactate were summarized into their respective time-weighted averages (TWA) by dividing their respective area-under-the-curve [21] by the total minutes of monitoring. We determined the association between autoMAPSE_TWA_ and peak postoperative ProBNP and TnT by Spearman’s non-parametric correlation coefficient *rho*. This analysis was repeated for the other hemodynamic and echocardiographic variables. Within-patient changes in biomarkers were assessed with Wilcoxon’s signed rank test.

Patient characteristics are reported as the mean (standard deviation) or median [interquartile range] as appropriate. Normality was assessed by Shapiro–Wilk test and inspection of histograms. We did not perform any a priori sample size calculations. Missing data were not substituted. We defined statistical significance as a *P*-value < 0.05, using Stata 18.0 (StataCorp LLC) for all the analyses.

## Results

We included 50 patients (76% male) after on-pump cardiac surgery, of which 24 (48%) underwent isolated coronary artery bypass grafting (Table 1). Postoperatively, Ea/Ees-ratio and LVEF were preserved, while both autoMAPSE and manual MAPSE were reduced (Table 2). Avoidance of LV foreshortening in the 4C view using simultaneous biplane imaging (Multi-D®, GE Healthcare, Horten, Norway) was evident by the adequate LV lengths in 4C and 2C views (Table 2). Three patients (6%) had atrial fibrillation during the monitoring period.

**Table 1:** Patient characteristics (*N* = 50)

Age (years)	67 (8)
Male/female (n, %)	38/12 (76/24)
EuroSCORE II	2.6 (2.7)
Aortic cross-clamp time (min)	70 (32)
Cardiopulmonary bypass time (min)	97 (45)
LVEF, preoperative (%)	47 (12)
Manual MAPSE, preoperative (mm)	10.2 (2.6)
Myocardial infarction within 90 days (n, %)	15 (30)
Operation (n, %)	
Isolated CABG	24 (48)
Aortic valve replacement	20 (40)
Mitral valve replacement	4 (8)
Mitral valve repair	1 (2)
Surgery on thoracic aorta	6 (12)
Redo cardiac surgery	3 (6)
Propofol (mg/kg/hr)	2.4 [2.0–2.9]
Norepinephrine (mcg/kg/min)	0.03 [0.02–0.07]

Values are mean (SD), n (%), or median [interquartile range]. Manual MAPSE is from the anterior wall. *CABG* coronary artery bypass grafting, *LVEF* left ventricular ejection fraction, *MAPSE* mitral annular plane systolic excursion

**Table 2:** Hemodynamic and echocardiographic measurements in the ICU

Continuously monitored variables	All patients (*N* = 50)	Isolated CABG (*n* = 24)	Other surgeries (*n* = 26)	*P*-value
AutoMAPSE_TWA_ (mm)	7.2 (2.3)	8.0 (2.2)	6.3 (2.3)	0.01*
MAP_TWA_ (mmHg)	77 (7)	77 (7)	76 (7)	0.80
Heart rate_TWA_ (bpm/min)	75 (15)	77 (15)	73 (13)	0.37
CVP_TWA_ (mmHg)	7 (3)	7 (3)	6 (3)	0.56
Lactate_TWA_ (mmol/L)	1.0 (0.4)	0.9 (0.3)	1.1 (0.4)	0.07
ScvO_2 TWA_ (%)	67 (6)	68 (6)	66 (7)	0.36
PcvaCO_2 TWA_ (kPa)	1.0 (0.2)	1.1 (0.2)	1.0 (0.2)	0.07
Single echocardiographic measurements				
AutoMAPSE (mm)	6.8 (2.1)	7.3 (2.0)	6.2 (2.2)	0.04*
Ea (mmHg/mL)	2.1 (0.6)	1.9 (0.4)	2.3 (0.7)	0.01*
Ea/Ees-ratio	1.33 (0.6)	1.1 (0.4)	1.6 (0.6)	0.01*
Ees (mmHg/mL)	1.7 (0.6)	1.6 (0.7)	1.9 (0.6)	0.19
LVEF (%)	50 (15)	52 (15)	48 (16)	0.33
LV end-diastolic volume (mL)	119 (43)	116 (39)	121 (47)	0.92
LV end-systolic volume (mL)	63 (41)	63 (36)	63 (46)	0.31
LV length, 2C (cm)	9.0 (1.0)	8.9 (0.9)	9.1 (1.0)	0.46
LV length, 4C (cm)	9.1 (1.0)	9.2 (1.1)	9.1 (0.8)	0.63
Manual MAPSE (mm)	7.1 (2.2)	7.9 (1.9)	6.2 (2.2)	0.01*
Stroke volume (mL)	51 (15)	49 (19)	53 (9)	0.04*
Velocity–time integral (cm)	15 (4)	15.9 (5)	14 (3)	0.36

Values are mean (SD). Manual MAPSE is from the anterior wall. *AutoMAPSE* automatic measurements of mitral annular plane systolic excursion, *CABG* coronary artery bypass grafting, *CVP* central venous pressure, *Ea* effective arterial elastance, *Ees* end-systolic elastance, *LV* left ventricular, *LVEF* left ventricular ejection fraction, *MAP* mean arterial pressure, *MAPSE* mitral annular plane systolic excursion, *PcvaCO*_*2*_ central veno-arterial carbon dioxide difference, *ScvO*_*2*_ central venous oxygen saturation. *P*-value between isolated CABG and other surgeries by Mann–Whitney test

AutoMAPSE had a feasibility of 91% (1123 of 1231 total recordings). One patient was only monitored for 100 min due to a decision for early extubation, and a second patient was monitored for 40 min due to emergent reoperation. The feasibility was 95% (48 of 50 patients) for autoMAPSE_TWA_, 82% (41 of 50 patients) for non-invasive estimation of Ea/Ees-ratio, and 84% (42 of 50 patients) for LVEF. MAP ranged between 48 and 104 mmHg, and autoMAPSE ranged between 0.7 and 14.8 mm. There were no complications related to TOE.

### AutoMAPSE and ventriculoarterial coupling

**Fig. 2:**
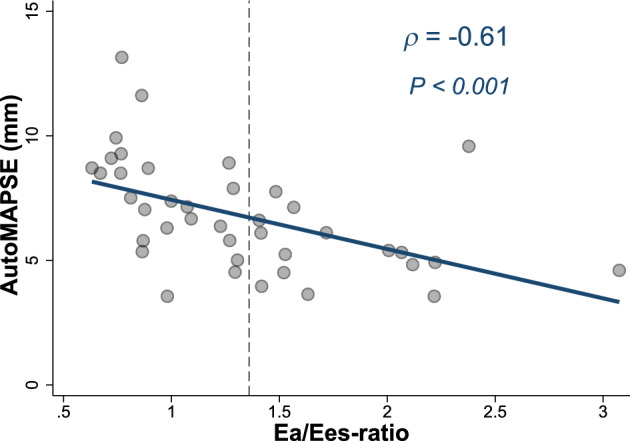
Correlation between a single measurement by autoMAPSE and non-invasive Ea/Ees-ratio. Dashed line at 1.36 indicates pathologic Ea/Ees-ratio. *AutoMAPSE* automatic measurement of mitral annular plane systolic excursion, *Ea* effective arterial elastance, *Ees* end-systolic elastance

AutoMAPSE was negatively correlated with Ea/Ees-ratio (Fig. 2), indicating a relation with VA coupling. Additionally, autoMAPSE correlated with Ees, ScvO_2_, and PcvaCO_2_, but not with Ea (Table 3). Similar correlations were observed between Ea/Ees-ratio and manual MAPSE (Supplementary Table 1).

**Table 3:** Spearman’s correlation coefficient between autoMAPSE and hemodynamic variables

	AutoMAPSE (single measurement)
Ea/Ees-ratio	− 0.61**
Ea	− 0.19
Ees	0.48**
Lactate	− 0.05
LVEF	0.49**
LVOT VTI	0.08
Manual MAPSE	0.79**
PcvaCO_2_	− 0.40*
ScvO_2_	0.45*
Stroke volume	0.28

Manual MAPSE is from the anterior wall, only. *AutoMAPSE* automatic measurement of anterior mitral annular plane systolic excursion, *Ea* effective arterial elastance, *Ees* end-systolic elastance, *LVEF* left ventricular ejection fraction, *LVOT VTI* left ventricular outflow tract velocity–time integral, *MAPSE* mitral annular plane systolic excursion, *ScvO*_*2*_ central venous oxygen saturation, *PcvaCO*_*2*_ central veno-arterial CO_2_ difference. * indicates P < 0.05; ** indicates P < 0.01

**Fig. 3:**
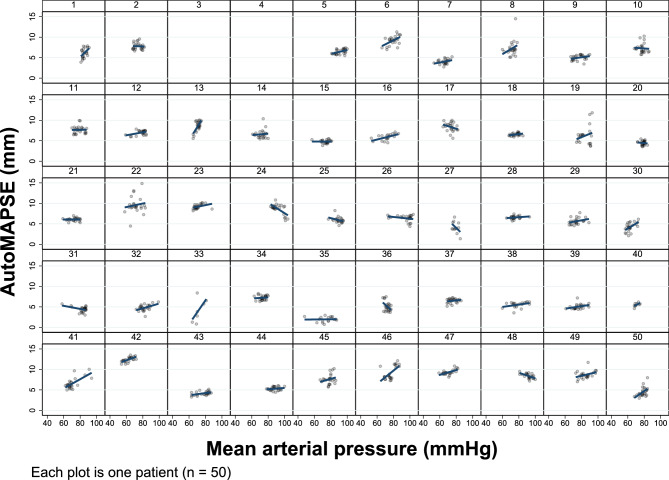
The correlation between autoMAPSE and MAP presented individually for each patient. The different individual slopes show how changes in MAP affect each patient’s autoMAPSE measurements differently. Patients number 3 and 4 did not have feasible measurements of autoMAPSE. *AutoMAPSE* automatic measurements of mitral annular plane systolic excursion, *MAP* mean arterial pressure

Notably, each patient exhibited an individual autoMAPSE/MAP-relationship (Fig. 3), indicating that patients respond very differently in terms of LV contractions when MAP changes. Of the 48 patients with feasible measurements, the individual slope was positive in 23 and negative in 25 (Fig. 3, *P* < 0.001). An intraclass correlation of 0.82 (*P* < 0.001) confirmed the large degree of patient heterogeneity.

### AutoMAPSE and postoperative cardiac biomarkers

TnT increased significantly from preoperatively to postoperatively (17 [11–61]) to 376 [226–580] ng/L, *P* < 0.001) and so did ProBNP (490 [141–1387] to 1530 [1031–3909] ng/L, *P* < 0.001). Overall, TnT peaked in the evening on the day of surgery, while ProBNP peaked in the evening on postoperative day 1.

**Fig. 4:**
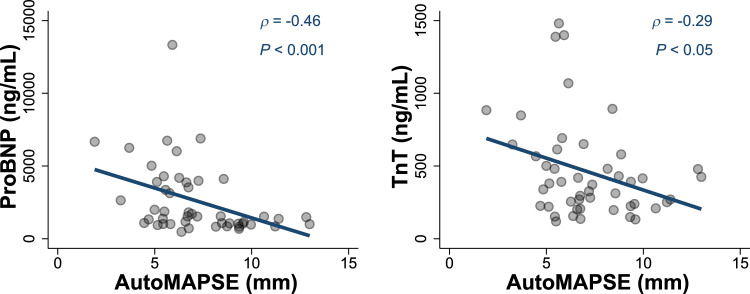
The relationship between the time-weighted average of continuous autoMAPSE measurements and the two biomarkers**. Left:** Peak postoperative ProBNP and continuous autoMAPSE (time-weighted average). **Right**: Peak postoperative TnT and continuous autoMAPSE (time-weighted average). *AutoMAPSE* automatic measurements of mitral annular plane systolic excursion

AutoMAPSE_TWA_ from the first two postoperative hours was inversely related to both peak postoperative ProBNP and peak TnT (Fig. 4). AutoMAPSE_TWA_ was the only hemodynamic variable that was associated with both of these biomarkers (Table 4). Other echocardiographic parameters were only associated with ProBNP at best (Table 4). Importantly, the commonly monitored variables (SV, cardiac output, MAP_TWA_, heart rate_TWA_, CVP_TWA,_ and RPP_TWA_) were not associated with either biomarker (Table 4).

**Table 4:** Spearman’s correlation coefficient between hemodynamic variables and cardiac biomarkers

Continuously monitored variables	Observations	ProBNP	TnT
AutoMAPSE_TWA_	48	− 0.46**	− 0.29*
CVP_TWA_	50	0.05	0.06
Heart rate_TWA_	50	0.11	− 0.11
Lactate_TWA_	50	0.00	− 0.02
MAP_TWA_	50	− 0.19	− 0.12
PcvaCO_2 TWA_	50	− 0.17	− 0.05
RPP_TWA_	50	− 0.04	− 0.21
ScvO_2 TWA_	50	− 0.14	− 0.30*

Single echocardiographic measurements			
AutoMAPSE (single measurement)	44	− 0.33*	− 0.10
Cardiac output	45	− 0.00	− 0.19
Ea/Ees-ratio	41	0.26	0.06
Ees	41	− 0.23	− 0.02
LVEF	42	− 0.31*	− 0.16
Manual MAPSE	44	− 0.39*	− 0.18
Stroke volume	45	− 0.06	− 0.18

Manual MAPSE is from the anterior wall, only. Stroke volume and cardiac output are measured by Doppler

*AutoMAPSE* automatic measurements of mitral annular plane systolic excursion, *CVP* central venous pressure *Ees* end-systolic elastance, *LVEF* left ventricular ejection fraction, *MAP* mean arterial pressure, *MAPSE* mitral annular plane systolic excursion, *PcvaCO*_2_ central veno-arterial CO_2_ difference, *ProBNP* N-terminal pro B-type natriuretic peptide, *RPP* rate-pressure product, *ScvO*_2_ central venous oxygen saturation, *TnT* high-sensitivity troponin-T, *TWA* time-weighted average. *indicates P < 0.05; **indicates P < 0.01

## Discussion

Our study shows that autoMAPSE reflects VA coupling, and that the interaction between MAP and VA coupling differed substantially between patients. Additionally, our findings show that continuous monitoring using autoMAPSE in the first two postoperative hours was associated with both the postoperative peak ProBNP and peak TnT in the subsequent 24 h. Although the strength of this latter association was limited, it still appeared better than conventional hemodynamic monitoring and single echocardiographic measurements.

The clinical implication of these findings is that autoMAPSE can potentially serve as a complementary tool to conventional hemodynamic monitoring and management. Currently, the focus is mainly on cardiac output and arterial pressure [22], which reflect the cardiac energy output. Although energy output is vital for organ perfusion, hemodynamic therapies that aim to increase energy output may sacrifice cardiac energy balance by increasing cardiac energy consumption [23]. Energetic imbalance can lead to LV dysfunction, biomarker release, and ultimately heart failure with cardiogenic shock. One way of optimising the trade-off between energy output and consumption is through an optimisation of the VA coupling [24]. Thus, autoMAPSE may potentially serve as a monitoring tool that reflects cardiac energetics more accurately than conventional hemodynamic variables. This hypothesis is supported by autoMAPSE_TWA_ showing a stronger correlation with postoperative biomarkers than any other hemodynamic variable measured in this present study. The potential usefulness of monitoring VA coupling in intensive care patients is further supported by the heterogenous autoMAPSE/MAP-relationships, which indicate that the effects of MAP on VA coupling are not predicted by arterial pressure alone.

### AutoMAPSE and ventriculoarterial coupling

The correlation between VA coupling and autoMAPSE found in the present study concords with previous studies using other echocardiographic parameters of systolic function. Indeed, the relationship between VA coupling and parameters for systolic function has been shown to hold for LVEF [3, 4] and several parameters of right ventricular systolic function [25]. For VA coupling and LV longitudinal function, animal studies have shown that global longitudinal strain is highly correlated with invasive measurements of Ea/Ees-ratio [26]. This latter finding also concords with the findings of our present study because global longitudinal strain and MAPSE are strongly correlated [27]. These findings indicate that all parameters of systolic function in essence are influenced by VA coupling.

The two approaches for assessing the relation between autoMAPSE and VA coupling were complementary. In the first approach, we found a moderate correlation between autoMAPSE and VA coupling. However, although we used the recommended method for clinical assessment of VA coupling [4], this method still has important limitations: In Chen’s original report [18], the method for non-invasive estimation of Ees had an excellent correlation and a low bias when compared to invasive measurements; however, the Bland–Altman plot showed somewhat wide limits of agreement. Therefore, the validity of this non-invasive method has been challenged [28]. In the second, more indirect approach, we correlated autoMAPSE with MAP for each individual patient. Notably, the individual autoMAPSE/MAP-relationship per se is not necessary for assessing VA coupling. Rather, this approach presumed that autoMAPSE reflected VA coupling and confirmed that the changes in MAP affected VA coupling when assessed by autoMAPSE; otherwise, a significant relationship between MAP and autoMAPSE would be unlikely. Thus, the combined findings from the two approaches indicate that autoMAPSE reflects VA coupling.

**Fig. 5:**
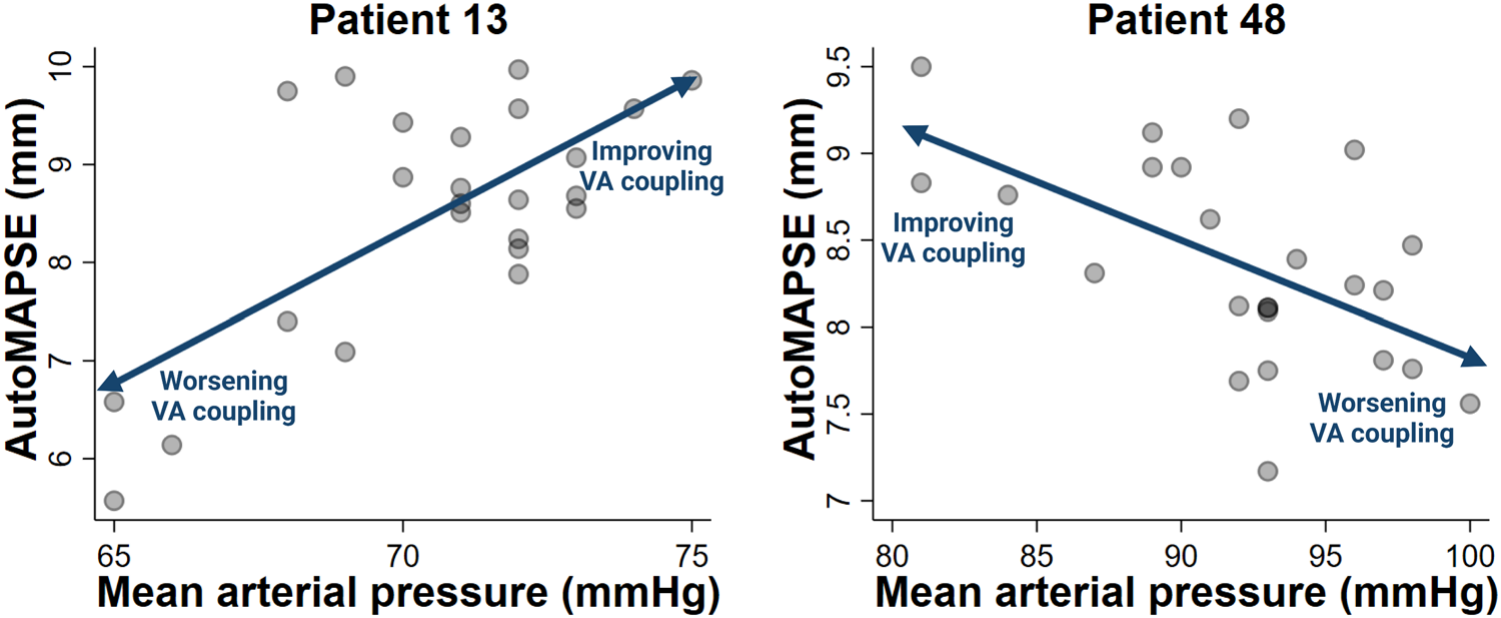
Two case examples from our data illustrating positive and negative autoMAPSE/MAP-correlations. **Patient 13**. This patient underwent urgent surgery for endocarditis and was initially hypovolemic. After blood transfusions, both autoMAPSE and MAP improved (positive slope), indicating an improvement in VA coupling after correcting hypovolemia despite a higher arterial blood pressure. An Ea/Ees-ratio of 0.7 corresponded to a relatively high MAP (74 mmHg) and autoMAPSE (9.9 mm), which indicates that VA coupling was optimized when both autoMAPSE and MAP were relatively high for that patient. Thus, autoMAPSE presumably tracked the improvement in VA coupling during volume resuscitation. **Patient 48**. This patient suffered an acute myocardial infarction and required urgent coronary artery bypass grafting. AutoMAPSE decreased during periods of higher MAP (negative slope), possibly due to reduced contractile reserve after myocardial infarction. An Ea/Ees-ratio of 1.3 corresponded to a relatively high autoMAPSE (8.9 mm) and a relatively low MAP (84 mmHg), which indicates that VA coupling was already near-pathological at this arterial pressure. In periods of higher MAP, decreasing autoMAPSE likely revealed worsening VA coupling. *AutoMAPSE* automatic measurements of mitral annular plane systolic excursion, *Ea* effective arterial elastance, *Ees* end-systolic elastance, *MAP* mean arterial pressure, *VA* ventriculoarterial

The different slopes of the individual patients’ autoMAPSE/MAP-relationship are presumably explained by different physiological mechanisms and pharmacologic treatments affecting VA coupling. We illustrate the clinical relevance of this autoMAPSE/MAP-relationship through two case examples from our data (Fig. 5 with explanations in legend).

For the positive slopes, increasing MAP with increasing autoMAPSE may reflect an improvement in VA coupling from improved Ees or decreased Ea. Admittedly, increasing MAP with decreasing Ea may seem counter-intuitive, but several studies have demonstrated this relationship during fluid infusion for relative hypovolemia [29–31]; a mechanism that seems to be the case in our first case example (Fig. 5, patient 13). As mentioned, improved Ees would also improve VA coupling and increase MAP, thus serving as an alternative mechanism for the positive slopes in the autoMAPSE/MAP-relationship. For the negative slopes, increasing MAP with decreasing autoMAPSE could reflect worsening VA coupling from increased Ea in patients with poor contractile reserve (Fig. 5, patient 48). Finally, a flat slope would likely represent sufficient contractile reserve, where an increase in MAP and Ea would be met with an increase in Ees, thereby rendering autoMAPSE and VA coupling unchanged. Thus, even though several physiological mechanisms may explain the different slopes of the individual autoMAPSE/MAP-relationship, the net effects on VA coupling may still be monitored by autoMAPSE.

We hypothesise that, in certain patients, there may be a reverse U-shaped autoMAPSE/MAP-relationship, where critical hypotension and hypertension can cause VA uncoupling and reduce autoMAPSE through mechanisms such as supply/demand-ischemia and pressure overload, respectively. These critical MAP thresholds are likely different for each patient and determined by the interactions of several other physiological factors, where the net effects on VA coupling could potentially be detected by continuous monitoring using autoMAPSE. However, our observational data could not test this hypothesis because MAP is controlled by the clinical team using vasoactive agents. Future studies should explore whether continuous autoMAPSE can guide individual MAP targets for optimal VA coupling in high-risk patients.

### AutoMAPSE and postoperative cardiac biomarkers

Another important finding was the association between autoMAPSE and the two cardiac biomarkers, which supports the hypothesis that autoMAPSE reflects LV load and function. Previous validations of autoMAPSE used manual measurements of MAPSE as the reference method [11–14], but this comparison is limited by the measurement variability inherent to the reference method [32]. Comparing autoMAPSE with cardiac biomarkers as a third independent reference provides important evidence that autoMAPSE indeed reflects cardiac physiology [33].

A single autoMAPSE measurement did not correlate with peak postoperative TnT, whereas continuous autoMAPSE measurements did. This finding may indicate that continuous monitoring of MAPSE is a more representative measurement of the patient’s risk of biomarker release than any single measurement of LV function, likely because a single measurement can coincide with short-lived changes in LV function that poorly reflect the patient’s actual condition. Such short-lived changes during echocardiographic assessments can occur in clinical practice, emphasising the potential advantage of continuous monitoring.

### Limitations

Our study has several limitations. First, the study is observational and hypothesis-generating. The sample size is relatively small, which limits our ability to adequately adjust for confounding factors. The data was mostly limited to the two-hour monitoring period, and events after probe removal could be important for postoperative cardiac biomarkers, but these events were not recorded. Our study is further limited by the lack of hemodynamic interventions. Future studies should aim to determine whether interventions guided by autoMAPSE to optimise VA coupling can reduce postoperative biomarker release and improve patient outcomes. Second, our calculation of Ees may be inaccurate in some patients; we estimated Ees using TOE, LVEF by Simpson’s biplane method and radial artery pressure, whereas Chen et al. estimated Ees using transthoracic echocardiography, LVEF by Teichholz’ method and arterial pressure by oscillometry [18]. Furthermore, the radial artery pressure deviates somewhat from that of the central aortic pressure that is responsible for LV systolic wall stress and coronary perfusion [4, 31]. However, Ea as an index of arterial elastance has also been challenged [4, 34], and even invasive assessments of Ea/Ees-ratio have limited ability to fully describe the complex pathophysiology of heart failure [4, 35]. Third, we only monitored MAPSE from the anterior wall because both experimental [36] and clinical data [37–39] have shown that changes in MAPSE from any wall reflect changes in global LV function rather than changes in regional LV function of that wall. Thus, MAPSE cannot reliably detect regional myocardial ischemia, but the MAPSE of any wall can be affected by regional dysfunction that impairs global LV function. However, monitoring the MAPSE from one wall may inaccurately reflect global LV function in patients with a mobile apex or dyssynchroneous contractions. Still, the impact of these inaccuracies has not been well defined, especially when autoMAPSE is used for continuous monitoring of *relative* changes in LV function. Because the goal is to monitor relative changes, we have also refrained from proposing any cut-off values that dichotomise autoMAPSE to normal/abnormal. Finally, while none of our patients experienced complications from TOE, the possibility remains. Fortunately, severe complications, like bleeding or oesophageal perforation, are very rare [40]. Notably, the risks stem from probe insertion, flexion, and manipulation [41, 42], which we expect to have minimized by leaving the probe tip unlocked and focusing on hands-free imaging. The use of smaller probes for continuous TEE may further enhance safety over extended monitoring periods.

## Conclusion

AutoMAPSE reflects VA coupling, suggesting that autoMAPSE can be used to monitor and guide therapies that aim to optimize LV load and function. Furthermore, continuous monitoring using autoMAPSE during the first two postoperative hours reflected postoperative peak ProBNP and TnT detected in the following 24 h.

## Supplementary Information

Below is the link to the electronic supplementary material.

Supplementary file1 (DOCX 16 KB)

## Data Availability

The datasets used and analyzed during the current study are unavailable due to a lack of patient consent for this purpose.

## Abbreviations

2C: Midoesophageal two-chamber view
4C: Midoesophageal four-chamber view
AutoMAPSE: Automatic measurement of mitral annular plane systolic excursion
CVP: Central venous pressure
Ea: Effective arterial elastance
Ees: End-systolic elastance
LV: Left ventricular
LVEF: Left ventricular ejection fraction
MAP: Mean arterial pressure
MAPSE: Mitral annular plane systolic excursion
PcvaCO_2_: Central veno-arterial CO_2_-difference
ProBNP: N-terminal pro B-type natriuretic peptide
RPP: Rate-pressure product
ScvO_2_: Central venous oxygen saturation
SV: Stroke volume
TnT: High-sensitivity troponin-T
TOE: Transoesophageal echocardiography
TWA: Time-weighted average
VA: Ventriculoarterial
