# Supplementary material for: Monitoring systemic ventriculoarterial coupling after cardiac surgery using continuous transoesophageal echocardiography and deep learning
Source: J Clin Monit Comput. 2025 Jul 17;40(2):391–403. doi: 10.1007/s10877-025-01328-5 (PMC13053427; doi:10.1007/s10877-025-01328-5)
Supplement: Supplementary file 1 — Supplementary file1 (DOCX 16 KB) [file 10877_2025_1328_MOESM1_ESM.docx]

**Supplementary Table 1. Spearman’s correlation coefficient between manual MAPSE and ventriculoarterial coupling**

|  | Average MAPSE | | Anterior MAPSE | | Inferior MAPSE | | Septal MAPSE | Lateral MAPSE |
| --- | --- | --- | --- | --- | --- | --- | --- | --- |
| Ea/Ees | -0.67** | -0.73** | | -0.63** | | -0.70** | | -0.41** |
| Ees | 0.40** | 0.56** | | 0.42* | | 0.40* | | 0.29 |
| LVEF | 0.66** | 0.58** | | 0.57** | | 0.49** | | 0.56** |

Ea, effective arterial elastance; Ees, end-systolic elastance; LVEF, left ventricular ejection fraction; MAPSE, mitral annular plane systolic excursion. * indicates P < 0.05; ** indicates P < 0.01.
